# Scale-Space Feature Recalibration Network for Single Image Deraining

**DOI:** 10.3390/s22186823

**Published:** 2022-09-09

**Authors:** Pengpeng Li, Jiyu Jin, Guiyue Jin, Lei Fan

**Affiliations:** School of Information Science and Engineering, Dalian Polytechnic University, Dalian 116034, China

**Keywords:** image deraining, multi-scale, attention recalibration, feature fusion

## Abstract

Computer vision technology is increasingly being used in areas such as intelligent security and autonomous driving. Users need accurate and reliable visual information, but the images obtained under severe weather conditions are often disturbed by rainy weather, causing image scenes to look blurry. Many current single image deraining algorithms achieve good performance but have limitations in retaining detailed image information. In this paper, we design a Scale-space Feature Recalibration Network (SFR-Net) for single image deraining. The proposed network improves the image feature extraction and characterization capability of a Multi-scale Extraction Recalibration Block (MERB) using dilated convolution with different convolution kernel sizes, which results in rich multi-scale rain streaks features. In addition, we develop a Subspace Coordinated Attention Mechanism (SCAM) and embed it into MERB, which combines coordinated attention recalibration and a subspace attention mechanism to recalibrate the rain streaks feature information learned from the feature extraction phase and eliminate redundant feature information to enhance the transfer of important feature information. Meanwhile, the overall SFR-Net structure uses dense connection and cross-layer feature fusion to repeatedly utilize the feature maps, thus enhancing the understanding of the network and avoiding gradient disappearance. Through extensive experiments on synthetic and real datasets, the proposed method outperforms the recent state-of-the-art deraining algorithms in terms of both the rain removal effect and the preservation of image detail information.

## 1. Introduction

Images acquired outdoors in natural environments often show significant blurring and visual quality degradation due to rain streaks. Therefore, single image deraining has become a very important forward operation in many practical multimedia application scenarios [1,2,3]. With the continuous development of computer vision technology in recent years, the single image deraining problem has become a research hotspot [4,5,6,7]. Unlike video deraining, single image deraining has less referable information, so the design of deep learning network architecture for single image deraining is more challenging.

Previous traditional single image deraining methods often treat the deraining problem as an optimization problem, using physical filtering to recover clean rain-free images [8,9]. However, these methods can only remove certain rain streaks that obey a specific distribution and have limited recovery in the face of different types of rain streaks in real rainy images.

In recent years, researchers for the single image deraining problem have increasingly begun to apply deep learning approaches such as convolutional neural networks (CNNs) [10], generative adversarial networks (GANs) [11,12], and semi/unsupervised [13,14] learning to enhance performance. Compared with classical methods, these methods extract important feature information from a large amount of training data by exploiting the powerful learning ability of their deep networks to better solve single image deraining tasks. Research methods based on CNNs [15,16,17,18,19] have powerful image feature representation capabilities by learning the features of different rain streaks, then removing the rain streaks from the rainy images. GANs-based research methods have been proposed to further acquire image features in real rainy images that cannot be synthesized by the system, thus reducing the gap between recovered images and real clean images. Recently, researchers have proposed semi-supervised and unsupervised learning methods in order to solve the synthetic data limitation problem. Their direct extraction of rain streak features from real raw rain maps improves the generality of the rain removal method. Although the methods described above have shown positive outcomes in a variety of application environments, they still have many limitations. It is often very challenging to learn different types of rain streak features accurately and completely in the single image deraining process. The inaccurate estimation of rain streak features will lead to incomplete image recovery or lead to excessive removal of image feature information. Therefore how to fully explore rain streak features in spaces of different scales is important for deraining single images. In addition, many existing algorithms rarely try to recalibrate image feature information after image feature fusion, which leads to the poor preservation of image detail information.

To address the aforementioned problem, we present a Scale-space Feature Recalibration Network (SFR-Net) that integrates multi-scale feature extraction, attention recalibration, and feature aggregation for single image deraining. Specifically, the network uses downsampling to achieve a multiscale hierarchical parallel structure after extracting low-frequency feature information and then uses an integrated densely connected Multiscale Extraction Recalibration Block (MERB) to extract and characterize rich image detail features. The proposed Subspace Coordinated Attention Mechanism (SCAM) is embedded in MERB. The Coordinated Attention Recalibration Mechanism (CARM) is placed in the SCAM after cross-layer feature fusion to recalibrate the acquired features. For better information transfer and cross-layer multi-scale image feature fusion, the network is implemented by dense connectivity and upsampling, respectively. In summary, the following are our key contributions:

- We propose an SFR-Net based on densely connected multi-scale feature fusion to accomplish single image deraining. Its network architecture can learn richer image feature representations efficiently, from coarse to fine features.
- We propose CARM and SCAM, where CARM collects cross-channel and significant location feature information along the X and Y spatial directions, respectively. SCAM combines CARM and the Subspace Attention Mechanism (SAM) to recalibrate rain streaks features and reduce useless feature information transfers. The CARM and SCAM are designed to help the network retain spatial and background detail information better.
- We propose an MERB. The MERB uses dilated convolutions of different scales to extract feature information at different scales and fuses this information using Across–Up connection and Across–Down connection. The SCAM is placed in MERB after feature stitching to enable it to learn feature information better from the original image.

## 2. Related Work

In this section, we will briefly review some traditional and deep-learning-based methods for single image deraining, as well as some multi-scale learning applications in computer vision.

### 2.1. Single Image Deraining

Before 2017, scholars usually used model-driven methods based on a loss function between the rain and background layers and optimized it by prior knowledge. For example, Xu et al. [20] proposed a rain removal algorithm using a guided filter, which does not require pixel-based statistical information to detect rain streaks features. Zhang et al. [21] designed a Convolutional-Coding-based Rain Removal (CCRR) algorithm, which first decomposes a rainy image into a clean image and an image with only rain streaks, and then performs rain removal using a convolutional low-rank filter. In order to extract the image texture layer more efficiently while avoiding excessive smoothing of the image background layer, Gu et al. [4] decomposed a single image into two separate layers with different sparse representations to learn the large-scale feature structure and represent the small-scale texture information of the image.

In order to break the bottleneck of traditional methods, deep-learning-based single image deraining algorithms have been proposed one after another after 2017. Yang et al. [10] created the JORDER network to deal with overlapping rain streaks in a heavy rain environment. The JORDER obtains information such as rain streak regions during the detection phase and then uses this information to perform effective rain removal while losing certain texture details due to excessive information removal. In the same year, Fu et al. [1] further proposed a depth detail network (DDN) based on the previous work to avoid losing texture details; however, the method could not handle too many dense rain streaks. Following Yang and Fu et al., an increasing number of CNN-based approaches have been proposed. To alleviate the problem of difficult to reproduce deep network structures, Ren et al. [22] proposed a simple and effective progressive recursive rain removal network (PReNet). The lightweight pyramid network (LPNet) [23] consists of fewer parameters, thus making the network simple to better focus on the internal connections between rain streaks obtained at different scales. Squeeze-excitation network (RESCAN) [24] employs dilation convolution to obtain background information and uses recurrent neural networks to reshape rain features. GCANet [25] employs smooth dilation convolution instead of dilation convolution and incorporates contextual information to improve recovery. To promote the interpretability of the rain removal network, RCD-Net [26] uses a convolutional dictionary learning mechanism to encode the shape of the rain and a proximal gradient technique to design the optimization algorithm. Zhang et al. [12] applied conditional generative adversarial networks (CGAN) to the single-image rain removal problem in order to render better light, color, and contrast distributions for the rain removal results. Chen et al. [27] developed an effective unpaired single image deraining adversarial framework that explores the mutual properties of unpaired samples by means of double-contrast learning in the deep feature space, named DCD-GAN. However, the GAN-based approach is not good at capturing detailed information of images and thus has poor results for images with diverse rain streaks. Recently, semi-supervised and unsupervised learning methods [28,29] have been proposed to further improve the recovery performance of real rain images, and these methods learn features directly from real rain data as a way to improve the robustness of the methods.

### 2.2. Multi-Scale Learning

Multi-scale learning can help expand the deep network’s field of perception and thus is useful for improving the characterization of image geometric feature information [30]. Since rain streaks exhibit some self-similarity, obtaining correlation information at different scales can help improve the characterization of image features. Yang et al. [31] introduced a recurrent hierarchy enhancement network (ReHEN) to explore the association of adjacent stages step by step. Wang et al. [32] explored the deep cross-scale fusion network (DCSFN) for deraining. Jiang et al. [33] applied multi-scale and multi-level convolutional neural networks to fuse features to improve end-to-end single image deraining. Unlike the above methods, in this paper we perform multi-scale learning of image feature information by scale-space feature recalibration.

## 3. Proposed Method

In this section, we describe the overall framework of the SFR-Net proposed in this paper. In each subsection, we introduce the key modules of the network, including SCAM, CARM, MERB, and also describe the loss functions used in the experiments.

### 3.1. The Framework of SFR-Net

**Figure 1:**
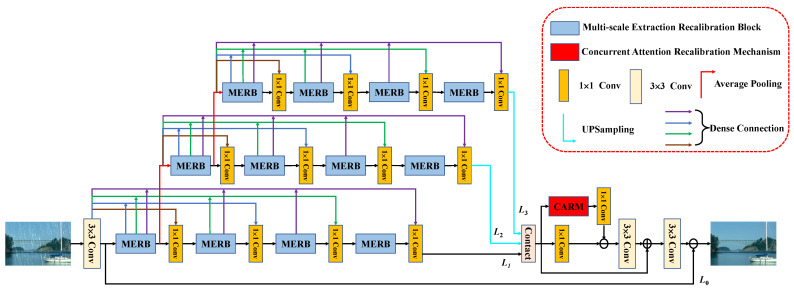
The proposed Scale-space Feature Recalibration Network architecture.

The overall structure of SFR-Net is shown in Figure 1. We propose an end-to-end network for clear recovery of images from rainy days, which consists mainly of MERB for feature extraction and CARM for recalibration. At the beginning of the network feature extraction, we learn the shallow original image features through a 3×3 convolutional layer whose output is $L_{0}={Conv}_{3\times 3}\left(I_{\mathrm{rain}}\right)$ and use it as the first MERB input. $L_{1}$, $L_{2}$, $L_{3}$ are the cross-layer outputs of feature extraction, and the overall network output $L_{out}$ can be derived by the following formulas:

(1) $$L_{cat}=Contact\left(L_{1},L_{2},L_{3}\right),$$

(2) $$L_{R1}=\left(Conv_{1\times 1}\left(CARM\left(L_{cat}\right)\right)-Conv_{1\times 1}\left(L_{cat}\right)\right),$$

(3) $$L_{R2}=Conv_{3\times 3}\left(L_{R1}\right)+L_{cat},$$

(4) $$L_{out}=L_{0}-Conv_{3\times 3}\left(L_{R2}\right),$$

where $L_{cat}$ denotes the output after cross-layer feature stitching. $L_{R1}$ and $L_{R2}$ are the outputs of the first and second feature calibration after feature stitching, respectively. CARM is the coordinate attention recalibration mechanism. $Conv_{n\times n}\left(\cdot \right)$ indicates the convolution kernel size is n×n convolution operations.

### 3.2. Subspace Coordinate Attention Mechanism

To further solve the single image deraining task, the important question is how to effectively gather and describe rain streak characteristics for removal. Although a deeper network is beneficial to extract the features of rain streaks, the ability to characterize the image features will gradually weaken with the transmission process as the depth of the network increases, as well as a vast quantity of duplicate feature information.

As a result, the resolution of these difficulties will have a direct influence on the quality of the recovered images. To eliminate the large number of redundant image information and extract more important image features, we created a parallel structure by merging the CARM and the SAM [34], which was inspired by the success of the computer vision attention mechanism.

**Figure 2:**
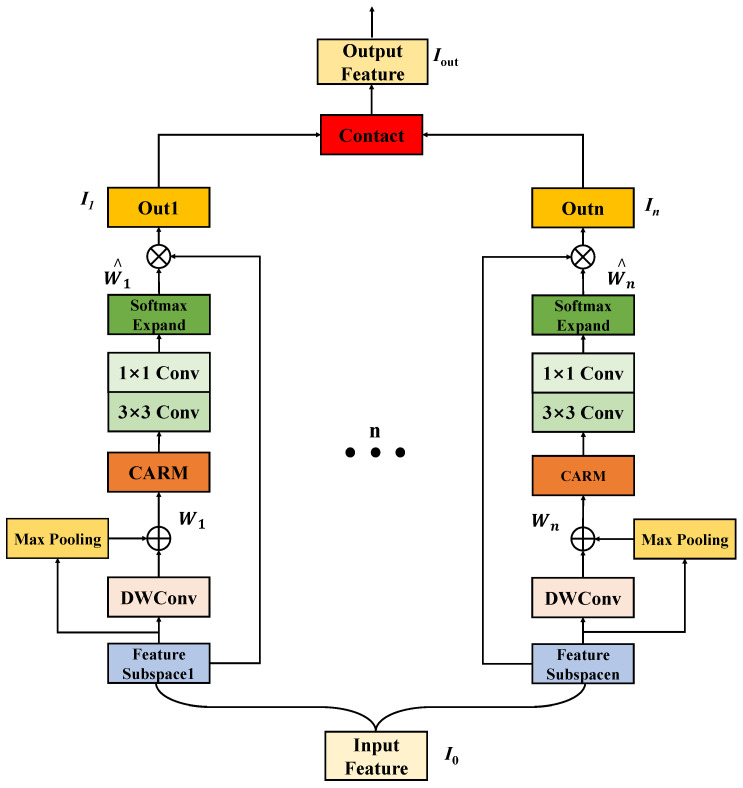
The proposed Subspace Coordinate Attention Mechanism..

The parallel coordination and subspace attention mechanism focuses on acquiring spatial and channel feature information and allows only features containing useful information to be further transmitted. As shown in Figure 2, the SCAM divides the input feature map ($I_{0}$) into n mutually exclusive subspaces: $\left[I_{1},I_{2},\ldots I_{\eta },\ldots I_{n}\right]$. We define $I_{\eta }$ as a set of intermediate feature maps, and its overall architecture can be formulated as:

(5) $$W_{\eta }=Maxpooling_{3\times 3}\left(I_{\eta }\right)+DWConv_{1\times 1}\left(I_{\eta }\right),$$

(6) $${\hat{W}}_{\eta }=Softmax\left(Conv_{1\times 1}\left(Conv_{3\times 3}\left(CARM\left(A_{\eta }\right)\right)\right)\right),$$

(7) $${\hat{I}}_{\eta }={\hat{W}}_{\eta }⊗I_{\eta },$$

(8) $$I_{\mathrm{out}\;}=Concat\left[{\hat{I}}_{1},{\hat{I}}_{2},\ldots {\hat{I}}_{\eta },\ldots {\hat{I}}_{n}\right],$$

where, in Equations (5) and (6), ${Maxpooling}_{3\times 3}$ is the maximum pooling operation with a kernel size of 3×3, ${DWConv}_{1\times 1}$ is the depthwise separable convolution with kernel size of 1×1, while ${Conv}_{1\times 1}$ and ${Conv}_{3\times 3}$ are the ordinary convolutions with kernel size 1×1 and convolution kernel size 3×3, respectively. We characterize the feature information learning across channels and spaces for each set of segmented subspaces, and ${\hat{W}}_{\eta }$ is the attentional feature map inferred from the feature information learning of an intermediate set of subspaces. In addition, we employ a softmax activation mechanism to ensure that ${\hat{W}}_{\eta }$ is a valid attentional weight that can recalibrate the feature information better. The CARM is the Coordinate Attention Recalibration Mechanism. Each set of feature maps will obtain a redefined feature map set ${\hat{I}}_{\eta }$ after Equation (7), where ⊗ is the element multiplication. The final output of SCAM is derived from Equations (5)–(7) together as Equation (8). Contact is the recombination of the feature maps of each group.

The SCAM is incorporated in each MERB to address information loss throughout the multi-scale feature gathering and transmission procedure. Feature refinement in SCAM is accomplished by the recalibration of feature information utilizing CARM and SAM.

**Figure 3:**
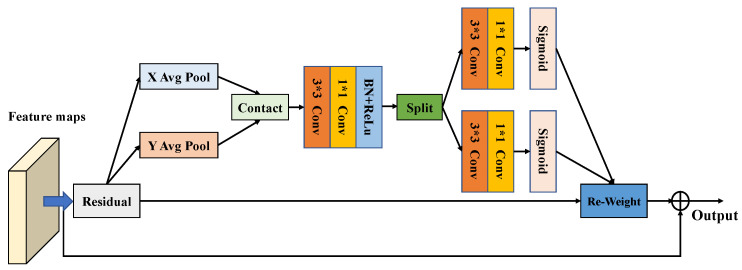
The proposed Coordinate Attention Recalibration Mechanism.

**Coordinate Attention Recalibration Mechanism:** In the real situation, the density of rain streaks is different on each channel of the image. However, many previous methods for single-image rain removal have not considered or cannot solve this problem well. Until the channel attention mechanism was proposed, researchers found that the channel attention mechanism could effectively obtain the weights of rain streak feature information on different rainy image channels. However, both the channel attention mechanism and the spatial attention mechanism proposed later [35] ignore the extraction of location-specific information when oriented to the image deraining problem. The location-specific feature information can help eliminate rain streaks better. Therefore, inspired by the coordinated attention mechanism [36], this paper designs a CARM, as shown in Figure 3, and embeds it in SCAM to enhance the network’s extraction capability for location-specific information to improve the network performance and accuracy. CARM differs from most previous attention mechanisms in that it collects cross-channel feature information along the X and Y spatial directions while also obtaining important perceptual information about direction and location.

### 3.3. Multi-Scale Extraction Recalibration Block

The Multi-scale feature extraction method effectively compensates for the lack of detail in image geometric features in deep networks, which combine image feature information at different scales. For further improved network representation, we propose MERB, which employs interlayer multiscale information fusion and allows information to be merged between features of different scales. Moreover, this structure ensures that all parameter layers receive input information, making it possible to learn the characteristic information of the original image better.

**Figure 4:**
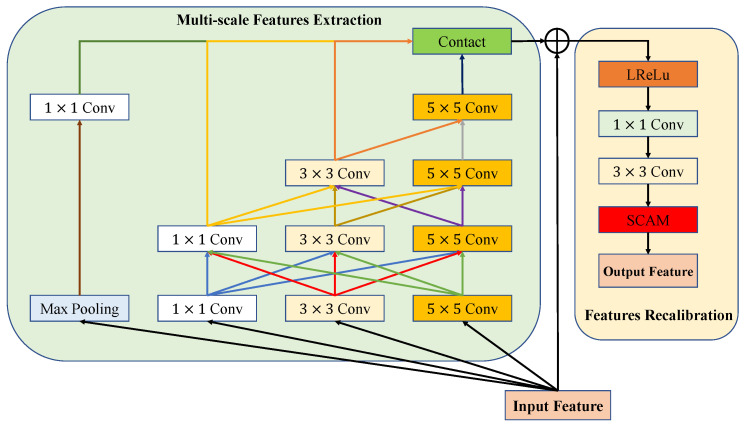
The proposed Multi-scale Extraction Recalibration Block.

Mathematical formulas can be used to describe the MERB in detail. According to Figure 4, the input of MERB is set to $F_{in}$, and the block can be formulated as follows:

(9) $$\begin{array}{cc} \; & F_{a}^{Maxpooling}=Maxpooling\left(F_{in};\theta_{a}^{Maxpooling}\right), \end{array}$$

where $F_{a}^{Maxpooling}$ denotes the output of the first layer after Maximum Pooling, and $\theta_{a}^{Maxpooling}$ means the hyperparameter formed by the Maximum Pooling:

(10) $$\begin{array}{cc} \; & F_{a}^{1\times 1}=Conv_{1\times 1}\left(F_{in};\theta_{a}^{1\times 1}\right), \end{array}$$

(11) $$\begin{array}{cc} \; & F_{a}^{3\times 3}=Conv_{3\times 3}\left(F_{in};\theta_{a}^{3\times 3}\right), \end{array}$$

(12) $$\begin{array}{cc} \; & F_{a}^{5\times 5}=Conv_{5\times 5}\left(F_{in};\theta_{a}^{5\times 5}\right), \end{array}$$

where $F_{a}^{n\times n}$ and $\theta_{a}^{n\times n}$ denote the output after the first layer of multiscale extraction and the hyperparameters formed by the first layer of multiscale extraction, respectively, and n×n is the size of the convolution kernel through which it passes. The $Conv_{n\times n}\left(\cdot \right)$ denotes the convolution operation. Further image features are extracted as follows:

(13) $$\begin{array}{cc} \; & F_{b}^{1\times 1}=Conv_{1\times 1}\left(\left(F_{a}^{1\times 1}+F_{a}^{3\times 3}+F_{a}^{5\times 5}\right);\theta_{b}^{1\times 1}\right), \end{array}$$

(14) $$\begin{array}{cc} \; & F_{b}^{3\times 3}=Conv_{3\times 3}\left(\left(F_{a}^{1\times 1}+F_{a}^{3\times 3}+F_{a}^{5\times 5}\right);\theta_{b}^{3\times 3}\right), \end{array}$$

(15) $$\begin{array}{cc} \; & F_{b}^{5\times 5}=Conv_{5\times 5}\left(\left(F_{a}^{1\times 1}+F_{a}^{3\times 3}+F_{a}^{5\times 5}\right);\theta_{b}^{5\times 5}\right), \end{array}$$

where $F_{b}^{n\times n}$ and $\theta_{b}^{n\times n}$ denote the output after the first layer of multiscale extraction and the hyperparameters formed by the first layer of multiscale extraction, respectively, and n×n is the size of the convolution kernel through which it passes. The $Conv_{n\times n}\left(\cdot \right)$ denotes the convolution operation. Similarly, we can obtain the output of the third and fourth layers as follows:

(16) $$\begin{array}{cc} \; & F_{c}^{3\times 3}=\left(Conv_{3\times 3}\left(F_{b}^{1\times 1}+F_{b}^{3\times 3}+F_{b}^{5\times 5}\right);\theta_{c}^{3\times 3}\right), \end{array}$$

(17) $$\begin{array}{cc} \; & F_{c}^{5\times 5}=\left(Conv_{5\times 5}\left(F_{b}^{1\times 1}+F_{b}^{3\times 3}+F_{b}^{5\times 5}\right);\theta_{c}^{5\times 5}\right), \end{array}$$

(18) $$\begin{array}{cc} \; & F_{d}^{1\times 1}=\left(Conv_{1\times 1}\left(F_{a}^{Maxpooling}\right);\theta_{d}^{1\times 1}\right), \end{array}$$

(19) $$\begin{array}{cc} \; & F_{d}^{5\times 5}=\left(Conv_{5\times 5}\left(F_{c}^{3\times 3}+F_{c}^{5\times 5}\right);\theta_{d}^{5\times 5}\right). \end{array}$$

As shown in Figure 4, MERB will enter the feature recalibration stage after passing the feature extraction. First, we perform feature stitching on the multi-scale feature information; then, we use the convolution kernel of size 1×1 and 3×3 for further deep extraction; and finally, we also introduce CARM to recalibrate the feature information. The final output of MERB is as follows:

(20) $$\begin{array}{cc} F_{out}=\; & CARM\left(\left(Conv_{3\times 3}\left(Conv_{1\times 1}\left(LReLu\right.\left(\;Contact\right.\right.\right.\right.\\ \; & \left(F_{d}^{1\times 1},F_{b}^{1\times 1},F_{c}^{3\times 3},F_{d}^{5\times 5}\right)+Fin\\ \; & \left.\left.\left.\left.\left.;\eta_{1}\right);\eta_{2}\right);\eta_{3}\right);\eta_{4}\right);\eta_{5}\right), \end{array}$$

where $F_{out}$ denotes the output of the MERB, and SCAM(·) indicates the Subspace Coordinate Attention Mechanism. $\left\{\eta_{1};\eta_{2};\eta_{3};\eta_{4};\eta_{5}\right\}$ indicates the hyperparameters of the MERB output.

### 3.4. Loss Function

In general, the recovered image obtained by the rain removal network should be as close as possible to the original clean image. In the training of the network in this paper, we use a hybrid loss function combining structural similarity loss (SSIM) [37] with MSE loss [38]. Specifically, SSIM loss is employed to measure structural similarity, which allows for greater preservation of high-frequency structural information. MSE loss is an excellent criterion for image restoration quality evaluation because of its ease of derivation, low computational cost, and clear physical meaning.

These two loss functions may be expressed as follows:

(21) $$L_{MSE}=\frac{1}{N}\sum_{i=1}^{N}{∥R-GT∥}^{2},$$

(22) $$L_{SSIM}=1-\mathrm{SSIM}\left(R,GT\right),$$

where $L_{MSE}$ and $L_{SSIM}$ represent MSE loss and SSIM loss, respectively. *R* is the image with rain, and GT represents the real rain-free image. Then the hybrid loss function in this paper can be represented by the combination of SSIM loss and MSE loss as:

(23) $$L=L_{MSE}+\lambda L_{SSIM},$$

where λ is a hyperparameter.

## 4. Experiments

In this section, details of the dataset, the experimental environment, and the parameter settings used in the experiments are described in detail. To demonstrate the good performance of the proposed method for the single image rain removal task, we perform quantitative and qualitative evaluations on synthetic and real datasets and compare the results with recent state-of-the-art methods. Finally, a complete ablation study is performed to demonstrate the significance of the key modules set in the proposed method.

### 4.1. Experimental Settings

#### 4.1.1. Datasets Setup

Four classical synthetic datasets are used in the training and testing experiments in this paper, and their specific composition is shown in Table 1. The Rain100L [10] dataset contains 200 training image pairs and 100 test image pairs in which there is only one type of rain streak in the rain image, which is a synthetic dataset for light rain scenes. The Rain100H [10] dataset consists of 1800 training image pairs and 100 test image pairs, in which the rain images consist of 5 types of rain streaks, which are synthesized for heavy rain scenes. The Rain800 [12] dataset consists of 700 training image pairs and 100 test image pairs, while the Rain1400 [1] dataset contains 14 types of rain streaks, from which 12,600 and 1400 image pairs are selected as training and test images, respectively. The real image dataset with rain is by Li et al. [39]. They proposed two existing datasets consisting of 185 and 34 real images, respectively.

#### 4.1.2. Evaluation Metrics

In the field of image processing, the effectiveness of the single image rain removal problem is often evaluated using the peak signal-to-noise ratio (PSNR) and the structural similarity index (SSIM). PSNR is an image evaluation index based on the error between corresponding pixel points. SSIM evaluates the similarity between two different images in terms of brightness, contrast, etc., and takes values in the range of 0–1. Therefore, the higher the values of PSNR and SSIM are when the rain image is better recovered by the deraining network. Because it is rare to produce a totally clean image in the actual world, it is difficult to quantify the recovery quality of a real image with rain. As a result, we will therefore visually evaluate the proposed network on real-world datasets.

#### 4.1.3. Implementation Details

For better extraction of image features, the number of MERBs is set to 12, as shown in Figure 1. In the training process, this design uses the Adam optimizer, where the default β1 and β2 parameters are 0.9 and 0.999, respectively. All experiments and tests in this paper use the PyTorch framework, and the graphics card is an NVIDIA Geforce RTX 3080Ti GPU (12G). To improve performance, the batch size is set to 32, the λ in the loss function is set to 0.2, and the initial learning rate is set to 0.001. For the Rain100L/H dataset, we train the network with 200 epochs and halved the learning rate every 50 epochs in the training process. For the Rain800/1400 dataset, we use 100 epochs, and the learning rate is halved every 25 epochs.

### 4.2. Experimental Results

#### 4.2.1. Results on Synthetic Datasets

In order to objectively evaluate the rain removal performance of the network structure proposed in this paper, we conducted extensive experiments on the synthetic datasets Rain100L, Rain100H, Rain800 and Rain1400. The experimental results are also compared with some mainstream advanced methods: GCANet [25], LPNet [23], RESCAN [24], DDN [1], JORDER [10], PReNet [22], RCD-Net [26], and DCD-GAN [27]. Table 2 shows the experimental quantification results of the different algorithms on the four synthetic datasets. It can be seen that the proposed method in this paper improves the PSNR and SSIM values compared to other methods, both for the small rain dataset with a single rainfall streak type and for the large rain dataset with more rainfall streak types. This indicates that the proposed network has better robustness and generalizability.

**Figure 5:**
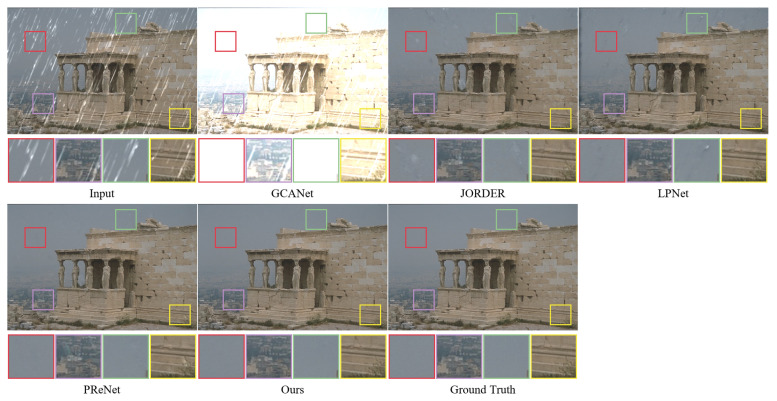
The performance of different methods on synthetic dataset (Rain100L).

**Figure 6:**
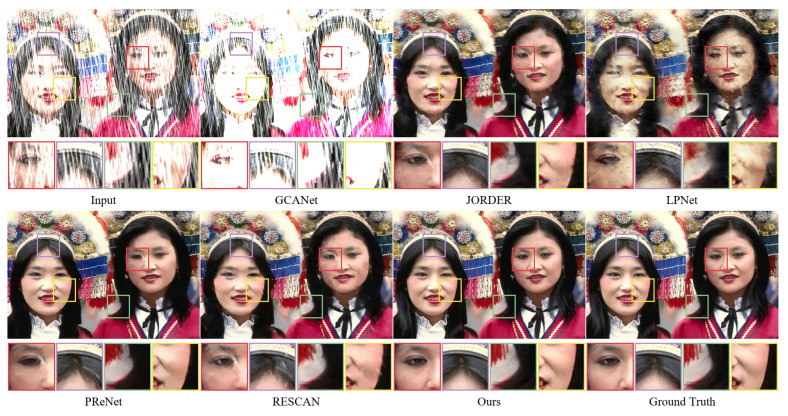
The performance of different methods on synthetic dataset (Rain100H).

We present various images for visual comparison in addition to the quantitative evaluation of the rain removal impact of a single image. As shown in Figure 5 and Figure 6, images derived from the small rain dataset Rain100L and the large rain dataset Rain100H are provided for visual comparison, respectively, and some areas of the images are selected and magnified in order to observe changes in image detail information. By observing the magnified local area, it can be found that although the GCANet algorithm is less effective in removing rain streaks, a large number of rain streaks remain; JORDER, LPNet, PReNet, and RESCAN remove a large number of rain streaks, but they all cause different degrees of background blurring and have certain defects in preserving the image background details. For example, the LPNet results on the Rain100L/H dataset all have varying degrees of texture distortion and blurring; the PReNet algorithm brings about loss of local details and color distortion. In comparison to the reference clean images, the method in this paper achieves good results and is able to remove the vast majority of rain streaks on a wide range of complex rain images.

Therefore, the SFR-Net designed in this paper greatly increases the interaction of feature information in scale-space by considering the interaction inside and outside the network, whether it is the skip connections outside the network or the cross connections inside the MERB. This design helps to better explore the correlation of image features in scale-space and can effectively remove rain streaks while retaining the background details on the synthetic datasets.

#### 4.2.2. Results on Real-World Datasets

**Figure 7:**
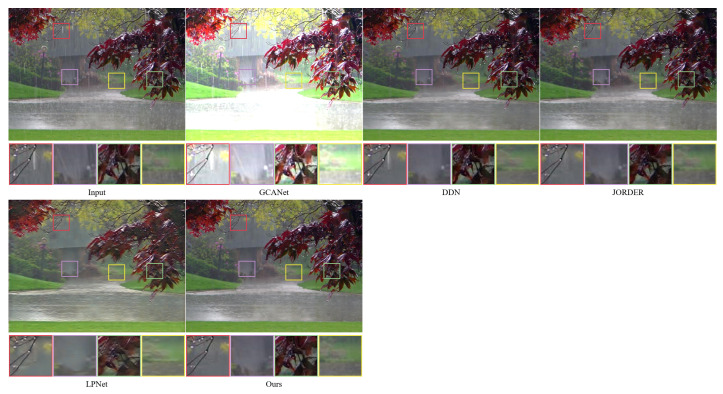
The performance of different methods on real world rainy dataset (Scene 1).

**Figure 8:**
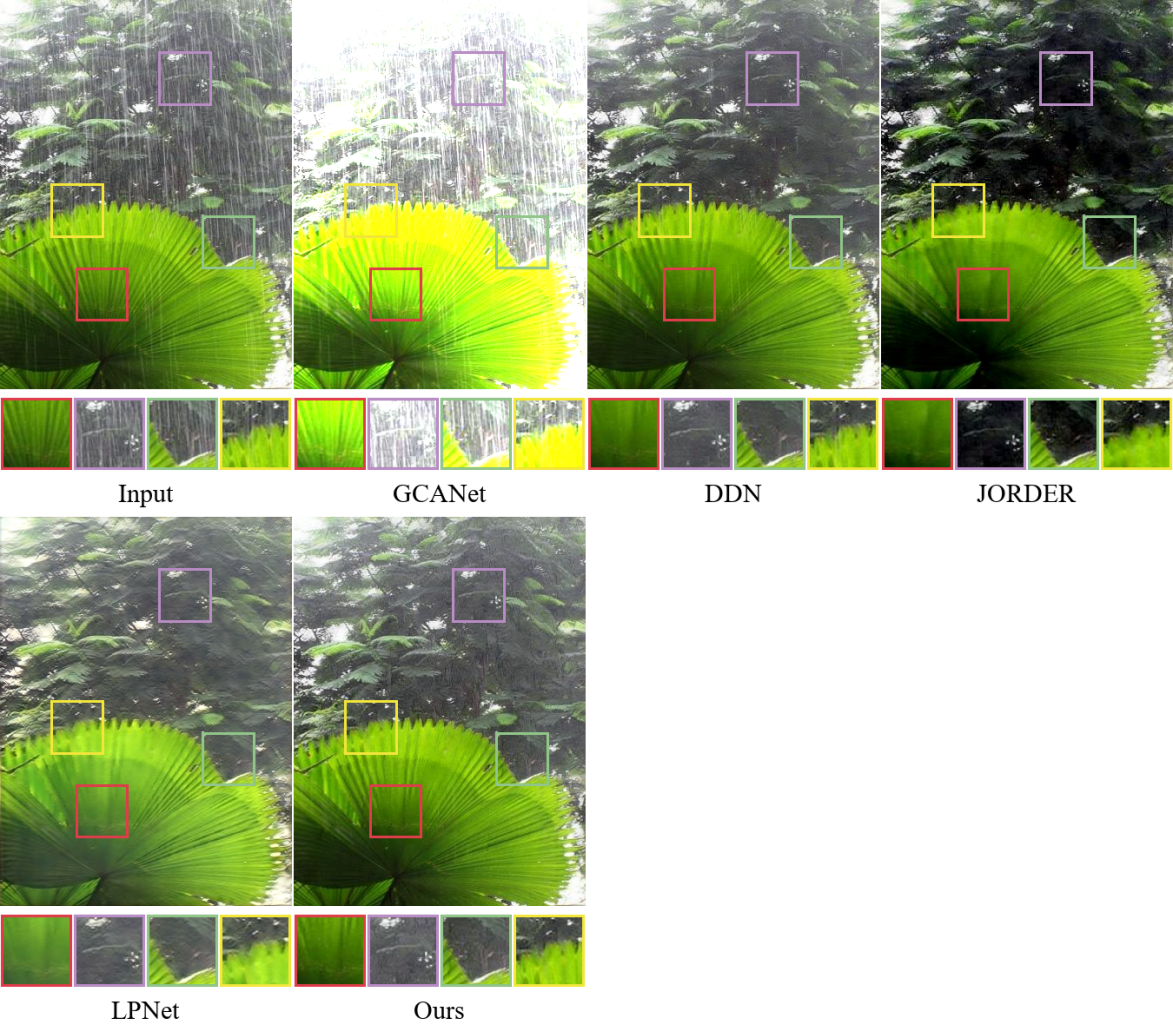
The performance of different methods on real world rainy dataset (Scene 2).

To further evaluate the rain removal effect of the method in real scenarios, we tested the proposed algorithm and the comparison algorithm in this paper on real datasets. For the fairness of the performance comparison, all methods use the weights of the pre-trained models obtained from the Rain100H dataset. As shown in Figure 7 and Figure 8, the method proposed in this paper produces a more natural and enjoyable recovered image compared to the mainstream methods. Specifically, it can be seen from the zoomed-in local details that GCANet has a large amount of rain streak residue and does not recover the image well; the DDN algorithm also has rain streak residue and poor feature processing of local regions, resulting in partial texture distortion and blurred image background; while the JORDER and LPNet algorithms also have blurring and color distortion.

The difference between the rain streaks feature information in the near and far scenes of rain images in the real datasets, as well as the excessive smoothing and more rain streak occlusion, may cause significant blurring and poor results after image recovery by the comparison methods. In this paper, instead of adding and fusing the feature information obtained from the hierarchical learning directly layer by layer, the feature information is stitched together using the residual projection at the end of the network for rainwater streak feature learning, which is helpful to improve the discriminative computing capability of the network. Therefore, by comparison, the method in this paper can more effectively remove rain streaks from real-world images with rain and retain more texture details.

### 4.3. Ablation Studies

To demonstrate the effectiveness and rationality of the network structure and experimental parameter settings of the SFR-Net proposed in this paper. We conducted a series of ablation experiments, all of which were performed under the same experimental environment and parameter settings. The uniform dataset used for the experiments was the Rain100L dataset.

#### 4.3.1. Ablation Study for the Proposed Scam

In this study, we present SCAM, which combines coordinated attention and subspace attention mechanisms. We investigated the effect of network deraining using the channel attention mechanism (CAM) [40], spatial attention mechanism (SPM) [35], SAM [34], and CARM, as shown in Table 3.

#### 4.3.2. Ablation Study of the Number of SCAM Subspaces

In order to investigate the effect of the number of subspaces in SCAM on the deraining effect, we conducted experiments with pairs of different numbers of subspaces. In particular, the number of subspaces is set to n∈{4,8,12,16}. The corresponding PSNR/SSIM results obtained are shown in Table 4. From Table 4, it can be found that increasing the number of subspaces can relatively obtain higher PSNR/SSIM values, which results in better extraction performance. However, after *n* = 8, the improvement in the PSNR seems to be limited. Therefore, after considering the balance between computational cost and performance, we choose *n* = 8 as the default parameter.

## 5. Conclusions

In this paper, we propose an SFR-Net to solve the single image deraining removal problem, which uses dense connectivity to achieve feature reuse and adequate propagation. To better acquire and characterize the feature information of rain streaks, a Multi-scale Extraction Recalibration Block is introduced to extract local and global features. In addition, this design applies a Subspace Coordinate Attention Mechanism to recalibrate image features by using coordinated attention recalibrate and subspace attention mechanisms to reduce useless features and preserve spatial and background information. Quantitative and visual intuitive results on both synthetic and real datasets show that the proposed approach outperforms the compared mainstream algorithms. However, the severe weather scenarios targeted by this design are only for rain, and the real application scenarios often include haze, rain, and snow in addition to rain. This design will be further upgraded towards a generalizable performance in a future work exploring inter-domain adaption to achieve domain migration and weight assignment of synthetic data using multi-source synthetic datasets for severe weather with complex noise and degradation factors, which better simulates severe weather image information and thus improves the robustness and generalization ability of the algorithm.

## Figures and Tables

**Table 1:** Descriptions of synthetic and real-world datasets.

Datasets	Rain100L	Rain100H	Rain800	Rain1400	Li et al. (Scene 1)	Li et al. (Scene 2)
Training Set	200	1800	700	12,600	-	-
Testing Set	100	100	100	1400	185	34
Type	Synthetic	Synthetic	Synthetic	Synthetic	Real-world	Real-world

**Table 2:** The Comparison Results On Synthetic Datasets.

Datasets	Rain100L (PSNR/SSIM)	Rain100H (PSNR/SSIM)	Rain800 (PSNR/SSIM)	Rain1400 (PSNR/SSIM)
Rainy	26.91/0.838	13.35/0.388	21.16/0.652	25.24/0.810
GCANet	31.70/0.932	24.10/0.814	-	27.84/0.841
LPNet	33.39/0.958	24.39/0.820	25.26/0.781	22.03/0.800
RESCAN	36.12/0.970	27.88/0.816	24.09/0.841	29.88/0.905
DDN	−	24.95/0.781	22.16/0.732	27.61/0.901
JORDER	36.55/0.974	22.79/0.697	26.24/0.850	27.55/0.853
PReNet	37.11/0.977	28.06/0.888	22.83/0.790	30.73/0.920
RCDNet	35.28/0.961	26.18/0.835	24.59/0.821	33.04/0.9472
DCD-GAN	38.12/0.970	27.88/0.816	25.61/0.813	30.75/0.920
**Ours**	39.67/0.988	30.90/0.912	30.60/0.904	33.98/0.955

**Table 3:** Ablation study on analysis of the proposed SCAM.

Framework	CAM	SPM	SAM	CARM	CAM + SAM	SPM + SAM	CARM + SAM
PSNR/SSIM	38.36/0.976	38.90/0.980	38.08/0.978	39.25/0.979	39.21/0.981	38.86/0.981	39.67/0.988

**Table 4:** Ablation study on number of subspaces for SCAM.

Metric	*n* = 4	*n* = 8 (Default)	*n* = 12	*n* = 16
PSNR/SSIM	39.08/0.9825	39.67/0.988.	39.20/0.9827	39.11/0.9826

## Data Availability

Not applicable.
